# Effectiveness of surface coatings containing silver ions in bacterial decontamination in a recovery unit

**DOI:** 10.1186/s13756-017-0217-9

**Published:** 2017-06-13

**Authors:** Rafael Manuel Ortí-Lucas, Julio Muñoz-Miguel

**Affiliations:** 1 Social Medicine and Public Health Department, Universidad Católica de Valencia San Vicente Mártir, C/ Espartero,7, Valencia, 46007 Spain; 2 Preventive Medicine Department, Hospital Clínico Universitario de Valencia, Valencia, Spain

**Keywords:** Antimicrobial activity, Silver ions, Risk assessment, Environmental contamination, Surface coating, Healthcare-associated infection

## Abstract

**Background:**

HAIs remain a frequent complication for hospitalised patients and pose a challenge that must be tackled by our health systems.

**Methods:**

Quasi-experimental study. In order to determine the antimicrobial effectiveness of surface coating agents containing silver ions (BactiBlock®) the degree of contamination of several surfaces in two ICU wards was compared.

The association between application of Bactiblock® and surface contamination was analysed using a relative risk (RR). Multivariate logistic regressions were performed for each product applied and each sampling location to adjust for the RR of the contamination of surfaces treated with Bactiblock® for the independent variables.

**Results:**

Surface contamination was observed in 31.5% of treated samples and 27.4 of untreated samples. Contamination was equally prominent on bedside Tables (38.7%), bed rails (38.4%) and sinks (38.3%), while the walls showed minimum contamination (2.6%). For beds under isolation protocols, contamination was higher (32.6%) than when no protocol was followed (26.5%) but the difference was not significant (*p* = 0.148). After stratification for application method and adjusting the multivariate models for period of the study and presence of isolated patients, the risk of contamination after the intervention increased when the coating agent was applied using a spray (OR = 1.79; 95% CI: 1.08-2.95, particularly in a dry and rugged surface such as that of bedside Tables (OR = 2.59; 95% CI: 1.22-5.52); and decreased when the product was applied using a roller on a smooth and continuously cleaned (or wet) Surface (OR = 0.42; 95% CI: 0.19-0.92).

**Conclusion:**

Coating of hospital surfaces with substances containing silver ions may reduce bacterial growth. However, the effectiveness of the coating agent is affected by application method and environmental conditions and the type and cleanness of the surface.

## Background

Despite efforts to promote infection control and prevention protocols [1], healthcare associated infections (HAIs) remain a frequent complication for hospitalized patients and a challenge for healthcare systems, not only because they result in high morbi-mortality rates, but also because of the additional associated costs. For over 20 years, the microbiome has been considered the main source of nosocomial infection, followed by horizontal transmission between patients and healthcare workers (20-40%), and the environment (20%) [2]. However, the role of environmental contamination may be more relevant than previously thought. The importance of surface contamination in patient settings is one of the most controversial aspects of HAI control. Microorganisms can survive in the environment for different amounts of time. Certain pathogens such as Meticillin-resistant *Staphylococcus aureus* (MRSA), *Acinetobacter spp*. or *Pseudomonas aeruginosa* are capable of surviving for up to several months [3]. Besides the microorganism itself, the variability of their survival times on inanimate surfaces is conditioned by the nature of the surface, humidity, temperature, the cleaning procedure used, and the use of disinfectants as well as their intensity [4]. However, the lack of surface cleanness-control standards for nosocomial pathogens and the scarcity of experimental studies in this area limit the scientific evidence for a link between environmental contamination and the incidence of HAIs. Thus, surface colonisation studies are only recommended in outbreak scenarios [5].

Healthcare environment decontamination is not limited to the use of water, detergents, and disinfectants because conventional cleaning practices are not sufficient to guarantee decontamination [4, 6]. Although a surface may look clean, further testing often reveals the presence of organic remains and nosocomial pathogens [7]. Moreover, the efficacy of an agent depends on its correct use and the thoroughness of the cleaning and decontamination process [8, 9]. Recently, healthcare professionals have pointed out a direct relationship between healthcare budget cuts and the prevalence of HAIs [10]. In the context of preventive medicine, this means that the application of hygiene protocols has become more limited and, as a result, high-touch surfaces can become a reservoir for microorganisms [6, 7] thus facilitating the proliferation of infection and colonisation through indirect contact between healthcare workers, patients, and family members [11, 12]. Besides questioning the quality of traditional cleaning and disinfection methods, healthcare practitioners are now also becoming increasingly concerned about multi-drug resistant organisms related to HAIs, which are on the rise [13]. This has renewed interest in the prevention of these infections and strategies for their control in healthcare facilities [14]. New approaches have been proposed, such as employing novel disinfectants, steam, automated aerosol dispensers, or the use of surfaces with antimicrobial properties. However, these techniques must be properly evaluated before their implementation as means to prevent infection during the provision of healthcare services [12].

The development of antimicrobial surface coatings may play an important role in HAI reduction. These active surfaces are capable of reducing microbial counts compared to regular, untreated surfaces, and could reinforce hygiene in clinical environments [15]. Metals with biocidal properties have been used successfully to treat surfaces in the healthcare context [16]. Several different antimicrobial technologies are now available for the control of pathogenic microorganisms. For example, both in vivo [17] and in vitro [18] studies have proven the effectiveness of metallic copper as an antimicrobial which can reduce bacterial count. Similarly, the antimicrobial effect of silver is also well known and has been scientifically demonstrated [19]. A pilot study carried out by the Heart of England NHS Foundation Trust in the U.K. revealed that treating surfaces with ionic silver can reduce the levels of contaminant bacteria in healthcare environments by up to 95.8% [12, 20]. The innovation in the surface coating agent used in this study is its incorporation into ionic laminar clays before these are added to a polymer matrix [15]. These nanoclays distribute the silver ions evenly through the matrix, thus conferring the material consistent antimicrobial activity throughout the entire surface [21], as well as assuring uniform liberation of the product over time. Given both the demonstrable value of the minimum inhibitory concentration of nanosilver particle additives in vitro, and their antimicrobial capacity as polymer-based products [15], this pilot study aimed to determine its effectiveness in a clinical practice context.

## Methods

### Study design

In this study of quasi-experimental design, the level of contamination of different surfaces treated with laminar nanoclay-based antimicrobial additives containing silver ions was compared to that of untreated control surfaces in a recovery unit (PACU).

*The intervention consisted in the application of* BB635A1 (Antimicrobial acrylic coating. 0.3% BactiBlock® 101 R4.47 and 0.3% Zinc Pyrithione) on bedside tables (melamine), walls (paint) and beds (metal railings). BB655A0 (Monolayer polyurethane coating. 0.3% BactiBlock® 101 R4.47) was applied on floors and sinks (metal) using a monolayer roll. Both product and application method were chosen depending on the surface that was to be treated. Since monitors have tactile surfaces they could not be included in this study. The PACU comprises two wards with similar design: si rooms per nurse control-unit which are both in daily transit. The study had a duration of 3 months, corresponding to the minimum time period the coating agent is expected to remain active, and was divided into two periods. In order to analyse the effectiveness of the intervention in the prevention of pathogen growth, one of the wards was treated with the coating agent while the other remained untreated as a control. There are no differences between the occupancy rate or type of patient in the different wards, as patients are checked in after surgery into any available bed. At the beginning of this study the unit had just been renovated. The whole unit was subjected to the same cleaning routine. Both wards were cleaned and disinfected following the standard protocols used at the hospital: the floor was cleaned following the ‘double bucket’ system with a disinfectant containing 15.05% alkyl dimethyl ammonium and 1.5% bis-(3-aminopropyl)-dodecylamine; surfaces were cleaned with a cloth and a quaternary ammonium-based disinfectant. All products were used according to their instructions and were applied consistently.

The variables “isolated patient” and “period” refer to infected or colonised patients and the endemic level of the PACU respectively. The floor surface was also treated with the coating agent, but no samples were taken due to its minimal role in HAI transmission. The recovery unit continued in normal operation at standard activity levels throughout the study.

### Application quality control

In order to evaluate the effectiveness of the product through time, 5 × 5 cm aluminum squares were placed on the walls and floors of the treated wards before applying the antimicrobial products according to the international standard JIS Z 2801 test (ISO 22196) for antimicrobial activity of plastics [22]. These were placed in the same locations in both symmetrical wards in surfaces of different intensity of use such as the floor at the entrance of the ward, cabinets and walls. Once the product was applied on the surfaces, these strips were collected and analyzed every 15 days.

### Microbiological sampling

Samples were taken from the surfaces weekly, on Tuesdays at 11 am, on 25 cm^2^ plate count agar (PCA), replicate organisms detection and counting (RODAC) dishes in order to detect the total aerobic bacteria count. To sample a surface, the dish was opened and the side with exposed agar was rubbed along the surface being studied while applying constant and uniform pressure and avoiding abrupt movements. Once the sampling process was completed, the dish was closed and labelled with a detailed identifier before being sealed in Parafilm. The surface was then cleaned with isopropyl alcohol so as to eliminate any agar residue. The sealed dishes were transported in a portable fridge, in aseptic conditions, from the Hospital to the microbiology lab. The researchers at this lab were blinded to the sample’s origin during the entirety of the study. After incubation for 48 h at 37 °C and 100% RH, the samples were analyzed and the results were expressed in terms of CFU/cm2, considering the area of the sample.

### Statistical analysis

Firstly, the mean contamination results for all sampled surfaces expressed in CFU/cm2 were compared. The frequency of contamination was estimated for the different surfaces surrounding the patient. A surface was considered to be contaminated when the CFU count exceeded 0.03 CFU/cm2, a conservative threshold when compared to other approaches to the evaluation of surface hygiene [23]. A simple analysis was carried out using JI-2 estimation (or Fisher test) and relative risk (RR) analysis in order to compare the contamination of surfaces in terms of their treatment (with or without intervention) and other explicative variables: product application method (spray or roller), surface type (rough or smooth), patient isolation-status, and the observation period (weeks 1-7 or first period vs. weeks 8-13 or final period). To adjust the RR of contamination for treated surfaces to these variables, multivariate logistic regression analyzes were carried out for the product application at every sample point (ENTER method). The odds ratio (OR) values were estimated with a confidence interval of 95%; a *p*-value of less than 0.05 was considered significant, and a *p*-value of less than 0.20 was required for inclusion of a variable in the multivariate model. SPSS 15.0 was the statistical package of choice.

## Results

A total of 155 samples were taken from tables, 154 from sinks, 151 from the beds, and 155 from the walls; 31.5% of the samples in the treated areas were contaminated, whereas 27.4% of samples from non-treated areas were contaminated, equivalent to a RR of 1.15 (95% CI: 0.90-1.47; *p =* 0.261). Contamination was higher on smooth surfaces (38.4%) such as the metal on the bed rails, than on rough areas (20.6%) including the wood on the tables and the walls (*p =* 0.000), giving a smooth/rough RR of 1.86 (95% CI: 1.43-2.41). Contamination was also higher when the product was applied with a roll compared to a spray (38.3% and 26.5%, respectively; *p =* 0.005), resulting in a roll/spray RR of 1.45 (95% CI: 1.13-1.86). It is noteworthy that samples from the first 7 weeks were more likely to be contaminated than samples from the second period of study (36.5% and 21.3%, respectively; *p =* 0.000), giving a RR of 1.71 (1.31-2.23). If the patient was isolated in the sampled room, contamination (32.6%) was slightly higher than if there had been no isolation (26.5%), but this difference was not significant (*p =* 0.148).

**Fig. 1:**
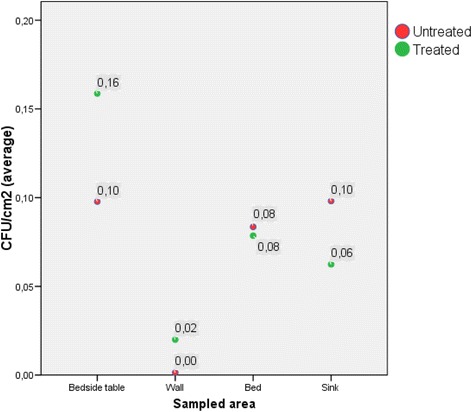
Level of microbial contamination on environmental surfaces (cfu/cm^2^), stratified according to intervention

When comparing the mean results of contamination for the different sample points (See Fig. 1), higher contamination was observed on treated beside tables than on untreated ones (*p* = 0.005). This effect was inverted in the sampled sinks, were treated sinks had lower mean contamination results. However these results were statistically non-significant (*p* = 0.13).

Similar results were obtained when the frequency of contamination as a dichotomous variable was analysed (Table 1). Interestingly, contamination was higher on bedside Tables (38.7%), sinks (38.3%), and beds (38.4%), than on the walls (2.6%; *p =* 0.000). The proportion of contaminated samples was higher from treated bedside tables, whereas there were no differences between treated and untreated bed rails; contamination was lower for treated sinks, although these differences were not statistically significant. There were statistically significant differences in the contamination of bedside tables both when isolated patients had been present in the room and during the first 7 weeks of study. Higher levels of contamination were also observed during the first half of the study for beds and sinks.

**Table 1:** Contamination frequency and simple analysis according to the sampling point and determining factors

Risk factor	Global		Bedside table		Bed		Wall		Sink	
	n (%)	*p*	n (%)	*p*	n (%)	*p*	n (%)	P _f_	n (%)	*p*
Intervention										
Yes	97 (31,5)	0.261	40 (51.3)*	0.001	29 (38.2)	0.949	3 (3.8)	0.317	25 (32.9)	0.172
No	84 (27,4)		20 (26.0)		29 (38.7)		1 (1.3)		34 (43.6)	
Surface type										
Rough (Wood or paint)	64 (20.6)*	0.000	60 (38.7)	-	-	-	4 (2.6)	-	-	-
Smooth (metal)	117 (38.4)		-		58 (38.4)		-		59 (38.3)	
Application Method										
Spray	122 (26.5)*	0.005	60 (38.7)	-	58 (38.4)	-	4 (2.6)	-	-	-
Monolayer roll	59 (38.3)		-		-		-		59 (38.3)	
Isolated patient										
Yes	56 (32.6)	0.148	23 (53.5)*	0.009	16 (37.2)	0.889	1 (2.3)	0.963	16 (37.2)	0.853
No	96 (26.5)		27 (30.0)		35 (38.5)		2 (2.2)		35 (35.6)	
Period										
Weeks 1-7	120 (36.5)*	0.000	40 (48.2)*	0.009	38 (46.9)*	0.021	0 (0.0)*	0.044	42 (51.2)*	0.000
Weeks 8-13	61 (21.3)		20 (7.8)		20 (28.6)		4 (5.6)		17 (23.6)	

* P < 0.05

f Denotes use of the Fisher test

Any of the determining factors described in Table 1 could have confounded our results, which resulted in a lower relative risk for the application of the product on dry, rough surfaces. Thus we prepared a logistic regression model for each type of sampled surface, adjusting for patient isolation and the study period as confounding factors. We explored the other possible explanatory variables mentioned above, but found that these resulted in no significant differences in the outcome.

**Fig. 2:**
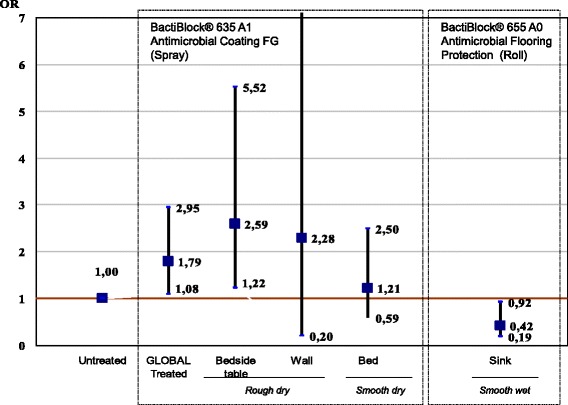
ORintervention for surfaces treated with spray / roll. (Logistic Regression: Sample point models adjusted for period and presence of isolated patients)

The OR for the multivariate model considering the global risk associated with spray application of the coating agent, was 1.79 (95% CI: 1.08-2.95). In specific models, according to the application method and sample point (Fig. 2), the intervention carries a relatively high risk of contamination for rough and dry surfaces like tables, which presented 2.6 times more risk (*p* < 0.05). This result also applies to the walls, although due to the low overall contamination incidence for this site, our model did not reach statistical significance and so *p* > 0.05. The result was also similar for the bed rails (smooth and dry surface), although the risk was lower (*p* > 0.05). Conversely, in the model that considered the global risk associated with the intervention using monolayer rolls, the intervention clearly had a protective effect for the sink (smooth and wet surface), with a nearly 60% decrease in contamination (OR = 0.42; 95% CI: 0.19-0.92).

## Discussion

Here, as a preventive alternative, we evaluated the in vivo effectiveness of BactiBlock® silver ion coatings. Although our results are coherent, because they show that there are higher contamination levels in the environment of the patient (bedside table, bed, sink), the effect of the silver coatings contradicted the beneficial effects previously reported in vitro [15]. However, before refuting the effectiveness of the coating agent, we must first evaluate any other factors that could explain this pattern. Our results suggest that the antibacterial effect of these products depends on the characteristics of the treated surface, especially its roughness and if it is generally wet or dry.

In vitro studies did not reveal any relevant differences in the efficacy of the coating agent applied as a spray or from a roll. Therefore, it is surprising that intervening by applying the spray on a wooden table (a dry and inherently rough surface) has a counterproductive effect by increasing contamination, whereas applying the same protocol to the sinks (a smooth and wet surface) by applying a roll, had the intended effect by reducing contamination. The same interventions on the metal bed rails (smooth and dry) or the wall (rough and dry) presented intermediate results, without any evidence of significant differences between these application methods.

The discrepancies between our results and those from previous studies could not be explained by the contamination evaluation procedure, the bacterial culture contact plate quality controls, or by differences resulting from the chosen sampling points because our results were consistent in every case. One limitation of our study was that not every microorganism that produced contamination was identified, however this does not explain the observed differences because all the samples were collected in the same environment using both an appropriate threshold to discriminate differential growth and an appropriate sample size. The fact that the occupancy rate was equally high in both wards and that no epidemical clusters were observed during the study lead us to believe that these factors played no significant role in the changes in contamination. Similarly, it does not appear that the differences we observed between surfaces are due to the development of natural resistance. It remains unclear if ionic silver can generate bacterial resistance [24, 25].

In contrast, it does appear that surface texture affects outcome. Surfaces like that of wooden tables or the plaster walls are rough, and their roughness is increased by the application of the spray. Corresponding with the decreased RR trend we observed for these materials, the metal surfaces of the bed rails and sinks are relatively smoother but their roughness is also enhanced by application of the spray, although the rails are dryer than the sinks, thus increasing their relative smoothness. Therefore, perhaps our results could be explained if the roughness of some surfaces prevents effective cleaning and limits the decontaminant effect of surface coatings. According to this hypothesis any dirt deposited on top of the antibacterial covering would inhibit the action of the silver ions present by creating a layer which blocks their direct contact with the deposited microorganisms. This phenomenon is not new; it is comparable with the effect observed in water pipelines when biofilms develop which favour the resistance of bacteria like *Legionella pneumophila* or *P. aeruginosa* to antimicrobials such as chlorine and other disinfectants [26].

It could be argued that contamination was lower in the sinks because they are continuously washed with tap water, however, it is well known that sinks can act as potential reservoirs for particular Gram-negative bacteria [24, 26–28] such as *Escherichia coli*, *Klebsiella spp*., or *Pseudomonas* spp. which are favoured in wet conditions, increasing their life expectancy to more than 1 year [3]. Nonetheless, if the dirt deposited on top of the antibacterial coatings is sufficient to render the antimicrobial action of the silver ions ineffective on rough ‘dirty’ (difficult to clean) surfaces, the near-continuous washing with water of the smooth (easy to clean) surface of the sinks would probably keep the coating ‘clean’, allowing it to be effective.

Although these products show in vitro biocidal activity against some microorganisms, it is important to assess their in vivo efficacy, because their effectiveness in real conditions depends on their proper application. This study suggests that the antimicrobial coating’s application method should avoid increasing the roughness of the treated surfaces, and that for the effective action of BactiBlock® (especially for rough surfaces), they should be wet-cleaned using a rubbing action in order to improve the liberation of silver ions from the product. This explanation is not novel in the context of medicine aimed at preventing HAIs. It is similar to the need to wash dirty hands so as to maximize the effects of hydroalcoholic solutions [29]. However, if confirmed, this information would be important in decision making, since it would help those controlling infections to better understand and manage the risks related to environmental contamination of health centres. Thus, the availability and proper application of new technologies is crucial when cleaning services and practices are insufficient or unable to reduce infection risks.

## Conclusions

Covering the surfaces of the hospital with silver ions may help to stop the increase in microorganisms, but further studies will first be required to confirm if the effect seen in previous studies is due to the product or to other concurrent factors present in these different environmental conditions such as the product type, application method, or whether the tested surface is dry or wet.

## Abbreviations

CFU: Colony forming units
HAI: Healthcare associated infections
ICU: Intensive care unit
MRSA: Meticillin-resistant *Staphylococcus aureus*
OR: Odds ratio
PCA: Plate count agar
RODAC: Replicate organisms detection and counting
RR: Relative risk
